# Advances in understanding and treating ADHD

**DOI:** 10.1186/1741-7015-9-72

**Published:** 2011-06-10

**Authors:** Kevin M Antshel, Teresa M Hargrave, Mihai Simonescu, Prashant Kaul, Kaitlin Hendricks, Stephen V Faraone

**Affiliations:** 1 State University of New York, Upstate Medical University, Department of Psychiatry and Behavioral Sciences, 750 East Adams Street, Syracuse, NY 13210, USA

## Abstract

Attention deficit hyperactivity disorder (ADHD) is a neurocognitive behavioral developmental disorder most commonly seen in childhood and adolescence, which often extends to the adult years. Relative to a decade ago, there has been extensive research into understanding the factors underlying ADHD, leading to far more treatment options available for both adolescents and adults with this disorder. Novel stimulant formulations have made it possible to tailor treatment to the duration of efficacy required by patients, and to help mitigate the potential for abuse, misuse and diversion. Several new non-stimulant options have also emerged in the past few years. Among these, cognitive behavioral interventions have proven popular in the treatment of adult ADHD, especially within the adult population who cannot or will not use medications, along with the many medication-treated patients who continue to show residual disability.

## Introduction

Attention deficit hyperactivity disorder (ADHD) is a neurobehavioral disorder that is defined by persistent and maladaptive symptoms of hyperactivity/impulsivity and inattention [1] (please see Table 1 for diagnostic criteria). People with ADHD often have serious impairments in academic, social and interpersonal functioning. ADHD is also associated with several comorbid conditions and disorders such as mood disorders, disruptive behavior disorders and learning disabilities. This review will review current conceptualizations of the causes of ADHD and advances in treatment, including discussions of stimulant and non-stimulant medication and cognitive behavioral therapy (CBT).

**Table 1:** DSM-IV^a ^criteria for attention deficit hyperactivity disorder

A. Either 1 or 2
1. Six (or more) of the following symptoms of inattention have persisted for at least 6 months, to a degree that is maladaptive and inconsistent with developmental level:
a. Often fails to give close attention to details, or makes careless mistakes in schoolwork, work or other activities
b. Often has difficulty sustaining attention in tasks or play activities
c. Often does not seem to listen when spoken to directly
d. Often does not follow through on instructions, and fails to finish schoolwork, chores or workplace duties (not due to oppositional behavior or failure to understand instructions)
e. Often has difficulty organizing tasks and activities
f. Often avoids, dislikes or is reluctant to engage in tasks that require sustained mental effort (such as schoolwork or homework)
g. Often loses things necessary for tasks or activities (for example, toys, school assignments, pencils, books or tools)
h. Is often easily distracted by extraneous stimuli
i. Is often forgetful in daily activities
2. Six (or more) of the following symptoms of hyperactivity/impulsivity have persisted for at least 6 months, to a degree that is maladaptive and inconsistent with developmental level:
a. Often fidgets with hands or feet or squirms in seat
b. Often leaves seat in classroom or in other situations in which remaining seated is expected
c. Often runs about or climbs excessively in situations in which it is inappropriate (in adolescents or adults, may be limited to subjective feelings of restlessness)
d. Often has difficulty playing or engaging in leisure activities quietly
e. Is often 'on the go' or often acts as if 'driven by a motor'
f. Often talks excessively
g. Often blurts out answers before questions have been completed
h. Often has difficulty awaiting turn
i. Often interrupts or intrudes on others (for example, butts into conversations or games)
B. Some hyperactive-impulsive or inattentive symptoms that caused impairment were present before 7 years of age
C. Some impairment from the symptoms is present in two or more settings (for example, at school/work or at home)
D. There must be clear evidence of clinically significant impairment in social, academic or occupational functioning
E. The symptoms do not occur exclusively during the course of a pervasive developmental disorder, schizophrenia or other psychotic disorder, and are not better accounted for by another mental disorder (or example,, mood disorder, anxiety disorder, dissociative disorder or personality disorder)

^a^*Diagnostic and Statistical Manual of Mental Disorders*, Fourth edition

### Understanding ADHD

The ADHD prevalence was once estimated to be 3 to 5% of school-age children [1], but more recent studies place the figure closer to 7 to 8% of school-age children [2] and 4 to 5% of adults [3]. Prevalence clearly varies, with risk factors including age, male gender, chronic health problems, family dysfunction, low socioeconomic status, presence of a developmental impairment and urban living [4]. The disorder is found in all countries surveyed, with rates similar to, if not higher than, those found in North America [5,6]. Differences across ethnic groups within North America are sometimes found, but seem to be more a function of social class than ethnicity [7]. Although diagnosed as a categorical disorder, ADHD may actually represent the extreme end of a normal continuum for the traits of attention, inhibition and the regulation of motor activity [8].

Current advances in cognitive neuroscience, neuroimaging, and behavioral and molecular genetics have provided evidence that ADHD is a complex neurobiological disorder. Many regions of the brain and several neurotransmitters have been implicated in ADHD. Biologically, the neurotransmitter dopamine has received considerable attention as being relevant to understanding ADHD. Neurologically, the prefrontal cortex seems to be relevant to understanding ADHD. The prefrontal cortex has a high requirement for dopamine, and plays a role in cognitive functions such as executive functions. The prefrontal cortex has many reciprocal connections with other brain regions, including the striatum (caudate nucleus, putamen), cerebellum and parietal cortex. Research has indicated that some of these brain regions are slightly smaller or have decreased activation in people with ADHD.

### Comorbid conditions

Pediatric ADHD commonly co-occurs with multiple psychiatric disorders including mood, anxiety and disruptive behavioral disorders [9,10]. Likewise, adult ADHD is also associated with diagnoses of comorbid mood, anxiety and substance-use disorder [11-17]. Comorbidity rates in adult ADHD do not differ as a function of gender [18]. ADHD in adults is not always comorbid with other concurrent psychiatric conditions, and some data suggest that 'uncomplicated' ADHD exists in about 20 to 25% of adults with ADHD [13].

### Current research

Research on ADH) has been published at an exponential rate during the past 30 years [19]. Within the past 3 years (2008 onwards), theories about the etiology of ADHD and therapies for it have evolved concurrently [20-23]. Psychopharmacological agents affecting catecholaminergic and α-2-adrenergic transmission continue to figure prominently in ADHD treatment [24,25]. Over the past 3 years, however, more attention has been paid to prescribing patterns [26-28], matching medication with patient characteristics [29], and factors that promote treatment adherence in pediatric [30-35] and young adult populations [36]. In the following section, current treatment options for both adults and children with ADHD will be discussed.

## ADHD treatments

There are both pharmacological and non-pharmacological treatments for ADHD for both children and adults. Pharmacological approaches to treatment are the most common, and typically consist of stimulant medication, such as methylphenidate, dexmethylphenidate, mixed amphetamine salts and lisdexamfetamine dimesylate (LDX). However, non-stimulants such as atomoxetine, clonidine and guanfacine have also been found to be efficacious in treating ADHD. In addition to medication, there are also non-pharmacological treatments. Many of the drugs discussed in this section are currently licensed for use in North America, but not in other countries.

Treatment for children with ADHD includes parent and teacher training in effective behavior-management techniques aimed at reducing the problem behaviors associated with ADHD. CBT is a skill-based approach commonly used for adults with ADHD and there are some preliminary data showing its efficacy.

### Stimulants

For most patients with ADHD, stimulants remain the first choice for medication management, as meta-analyses of existing research have shown that they are more efficacious than non-stimulant medications [37]. Various delivery mechanisms exist. Physicians may choose from a number of delivery mechanisms for these stimulants (liquid, sprinkle, tablet, capsule or patch); from active isomer, mixtures of active and less active isomers, or pro-drug; from and immediate-release, intermediate-release or extended-release formulations [38]. For both the methylphenidate and amphetamine families, there are arrays of choices, which enable practitioners to better tailor the duration of medication efficacy throughout the day to the needs of the individual (please see Table 2 for descriptions of stimulant options).

**Table 2:** Current pharmacological treatments approved for attention deficit hyperactivity disorder (ADHD)

Class	Generic name, formulation and brand name	Daily dosage	Duration	Mechanism of action	Common Side-effects
		**STIMULANTS**			
Methylphenidate	Immediate-release/short-acting (Ritalin, Methylin, Desoxyn)	Initial 5-18 mg; increase as needed until beneficial effects peak or unacceptable side effects develop Two to three times daily; can titrate as needed as long as beneficial effects are greater than side effects	3 to 6 hours	Blocks reuptake of D, N Release of D from storage vesicles	Appetite suppression, delay of sleep onset, abdominal pain, headache, rebound irritability, tics (motor, vocal), jitteriness
	Intermediate-acting (Metadate ER, Metadate CD, Methyllin ER, Ritalin LA, Ritalin SR)	One to two times daily	3 to 8 hours	Same	Same
	Extended release/long-acting (Concerta, Daytrana Patch)	Once daily (Patch left on for 9 hrs)	8 to 12 hours	Same	Same
Dexmethylphenidate	Short-acting (Focalin)	Two to three times daily; initial half that of IR MPH	4 to 5 hours	Same	Same
	Extended-release/long-acting (Focalin XR)	Once daily	8 to 12 hours	Same	Same
Amphetamines	Immediate-release/short-acting (Dexedrine, DextroStat, Adderall)	Initial dose half IR MPH; two to three times daily	4 to 6 hours	Release of D newly synthesized D; blocks reuptake of D, N	Same
	Intermediate-acting (Dexedrine spansule)	One to two times daily	6 to 10 hours	Same	Same
	Extended-release/long-acting (Adderall-XR)	Once daily	8 to 12 hours	Same	Same
Prodrug Amphetamines	Lisdexamfetamine (Vyvanse)	Initial 4 × IR MPH once daily	8 to 12 hours	Same	Same
		**NON - STIMULANTS**			
NRI	Atomoxetine (Strattera)	Initial 0.5 mg/Kg; Increase to 1.2-1.8 mg/Kg one to 2 times a day	18 to 24 hours	Blocks N reuptake at synapse	Sedation, GI irritability, palpitations, sweating, increased suicidal thoughts
		**ALPHA2 AGONISTS**			
Clonidine	IR Clonidine (Catapres)	Initial dose 0.05-0.1 mg at night; titrate to max 0.4 mg/per day	3 to 6 hours	Arousal at locus ceruleus by N inhibition	Sedation, Low blood pressure, rebound hypertension
	ER Clonidine (Kapvay)	Initial 0.1 mg qhs; titrate to max 0.4 mg qhs; once daily	12 to 24 hours	Same	Same
	Clonidine patch (catapres TDS)	Initial TTS-1 up to TTS-3	1 to 5 days	Same	Same
Guanfacine	IR Guanfacine (Tenex)	Initial 1 mg daily; titrate as needed up to 4 mg MDD; twice daily	12 to 24 hours	Same	Same
	ER guanfacine (Intuniv)	Initial 1 mg; up to 4 mg; once daily	~24 hours	Same	Same
		**ANTIDEPRESSANTS**			
NDRI	Buproprion	Initial: lesser of 3 mg/Kg/d or 150 mg; Maximum: Lesser of 6 mg/Kg/d or 450 mg; No singled does greater than 150 mg; 2 to 3 times a day	8 to 12 hours	N and D reuptake inhibition	Insomnia, decreased appetite, irritability, anticholinergic (dry mouth, GI etc.), decreased seizure threshold
	IR (Wellbutrin)			Same	Same
	ER (Wellbutrin SR)	Twice daily	12 to 24 hours	Same	Same
	(Wellbutrin XL)	Once daily	24 hours	Same	Same
					
SNRI's (Tricyclics)	Imipramine (Tofranil)	Initial: 1 mg/Kg/d; Maximum: Lesser of 4 mg/Kg/d or 200 mg; 1 to 2 times per day; obtain baseline EKG; Monitor serum levels	12 to 24 hours	N and Serotonin reuptake blockade	Sedation, Cardiac increase heart rate, arrhythmias, anticholinergic (dry mouth, GI, etc.), blurry vision

D = Dopamine,  N = Norepinephrine,  S = Serotonin,  IR = Immediate Release,  MPH = Methylphenidate,  mg/kg = milligrams/kilogram,  qhs = before bed

Research has continued to suggest that osmotic-release oral system (OROS) methylphenidate lessens ADHD symptoms throughout the day and has greater adherence, thought to be associated with the convenience of once-daily dosing [39-41]. Dexmethylphenidate extended-release (XR) and transdermal methylphenidate also offer this benefit. Dexmethylphenidate comes in capsules that can be opened and mixed with food, and has the earliest onset of efficacy of the long-acting preparations [42,43]. Transdermal methylphenidate bypasses the oral route entirely, and in short-term studies is associated with efficacy throughout the day, with improved family quality of life, and when carefully titrated, little effect on sleep [44-46]. Greater absorption of medication occurs when the patch is applied to the buttocks rather than to the subscapular area [47].

Comorbid anxiety was not found to affect stimulant efficacy in a recent study [48], and some studies suggest that treatment with stimulants can help to lessen the likelihood of other psychiatric comorbidities during adolescence [49], including cigarette use and substance abuse [50]. However, meta-analyses of stimulants and other ADHD medications in the treatment of ADHD comorbid with tic disorders concluded that supratherapeutic doses of dextroamphetamine should be avoided in this population. These studies also indicated that methylphenidate gave the best control for ADHD and that α-2-agonists produced the best improvement in both tics and ADHD [51,52].

The commonest side-effects of stimulants (decreased appetite, trouble with sleep onset) have also continued to receive recent research interest. Some research suggests that it is difficult to predict which children with ADHD will have adverse effects, based upon demographic and clinical characteristics [53]. Although rare, serious cardiovascular side-effects have been identified with stimulant use [54]; however, the common effects on blood pressure, heart rate and exercise parameters are usually of no clinical significance [55-57]. Consensus has been reached in the USA, Canada and Europe that routine electrocardiography screening and/or cardiology investigations are needed before starting stimulant use only in those with a positive family or personal cardiac history [58,59]. Likewise, no cytogenetic side-effects from stimulant use have been reported [60,61]. However, a recent review article has shown that treatment with stimulants in childhood modestly reduced expected height and weight [62]. These effects were dose-dependent and attenuated over time. The general consensus on cardiovascular side-effects at this point is that the short/medium term side-effects are usually of no clinical significance, but the long-term potential side-effects are less certain [63].

Within the past 3 years, concerns have continued to rise regarding the abuse of stimulants and/or drug diversion [64-66]. Some have suggested that in ADHD patients with conduct disorder or comorbid substance abuse, psychostimulants should be used with caution [67]. Long-acting stimulants are less prone to diversion, probably because extraction of active drug is more difficult, and for some, the delivery of drug to the brain is slower. The new stimulant pro-drug LDX offers some protection from these problems. LDX requires gut metabolism to reach its active form, and hence lessens the likelihood of abuse and overdose. The efficacy and side-effects of LDX are similar to the other long-acting stimulant preparations [68-72].

In summary, stimulant medication is often the first choice for medication management of ADHD. Research has shown that stimulant medication is an effective treatment for many of the symptoms associated with ADHD. However, there are some concerns about diversion of these medications for misuse and abuse, and some rare but serious cardiovascular side-effects can occur with the use of stimulant medication. In addition to stimulant medication, some non-stimulant medication has been shown to be efficacious for the treatment of ADHD (Table 2).

### Non-stimulants

Some children may not respond to stimulant medications, or may not be able to tolerate the stimulant medications due to side-effects (for example,, loss of appetite). Thus, several non-stimulant medications are also used for ADHD pharmacotherapy. Modafinil [73] and reboxetine [74,75] have both shown some promise in the treatment of ADHD. Drugs approved by the US Food and Drug Administration (FDA) for the treatment of ADHD include the selective norepinephrine reuptake inhibitor (SNRI), atomoxetine, a long-acting form of guanfacine, and a long-acting form of clonidine (Table 2). Clonidine and guanfacine have also been approved by the FDA for coadministration with stimulant medication.

Several reviews on the use of atomoxetine have been published recently [76,77], and studies in populations around the world have continued to confirm its efficacy [78-81], including for children with oppositional defiant disorder [82] or those who have received previous stimulant therapy [83]. When atomoxetine is administered once daily, some evidence suggests that morning dosing may be more efficacious, but evening dosing may be more tolerable [84]. In adolescents, doses in the higher ranges have been associated with greater long-term efficacy [85]. Because atomoxetine has been rarely associated with acute suicidality [86], it has been given a 'black box' warning. As with stimulant treatment, atomoxetine rarely completely normalizes behavior [87], but symptom improvement is often reflected in gains in social and behavioral function [88].

The α-2-adrenergic agonists clonidine and guanfacine have long been known to be of some assistance in treating ADHD [89]. Recently, once-daily extended-release guanfacine has proven effective in both short-term [90,91] and long-term studies [92,93]. Sedation is a common side-effect, which diminishes over time [94].

Response to single-agent treatment for ADHD often falls short of full remission. Recent studies have shown that adding clonidine to methylphenidate [95], extended-release guanfacine to stimulants [96], or OROS methylphenidate to atomoxetine [97] improved residual ADHD symptoms and was well tolerated. The FDA recently approved a long-acting form of clonidine to be used in monotherapy or as an adjunctive therapy to stimulant medications.

Treatment of ADHD can result in alleviation of comorbid depression, anxiety, oppositional defiant disorder and/or aggression [98]. However, when this is not the case, polypharmacy targeting each condition may have added benefit without unacceptable side-effects. Examples include use of atomoxetine or methylphenidate in children being treated for bipolar spectrum disorders [99,100] or borderline personality disorder [101], and atypical antipsychotics [102] or valproic acid preparations [103] for children with ADHD and aggression or bipolar disorder. For patients with autistic spectrum disorder, optimal results may require stimulants, SNRIs, antipsychotics and α-2-agonists [104].

A number of reports have been published about the use of alternative and complementary medicines in the treatment of ADHD. In a small study, traditional Chinese medicines were found to compare favorably with methylphenidate [105]. Positive results were claimed for gingko biloba [106], but this failed in head-to-head comparison with methylphenidate [107]. Short-chain fatty acids [108] and omega-3/omega-6 fatty acids [109] have not been found to be efficacious. A meta-analysis of neurofeedback treatment studies reported encouraging results that suggested the approach would be more effective for inattention and impulsivity than for hyperactivity [110]. Cognitive training paradigms [111] have also been forwarded as a potential treatment stratagem, although these data require further research before any meaningful conclusions can be reached. At this point, the consensus for most alternative and complementary therapies is that these therapies are best used as a complement to ongoing pharmacotherapy rather than as an alternative.

#### *Adult ADHD treatment*

The need for treatment in adults with ADHD has been debated in the past, with reports of suboptimal response, diversion and abuse. There is a growing body of research on the treatment, and recent years have seen the creation of evidence-based guidelines [112]. Meta-analysis of pharmacological agents for managing adult ADHD have shown that stimulant medications are more effective than non-stimulant medications. This is in line with pediatric ADHD, for which stimulants are also generally considered the front-line approach [113]. Also similar to pediatric ADHD, stimulants are generally considered the front-line approach for managing adult ADHD. Unlike pediatric ADHD, all the agents approved by the FDA for treating adult ADHD are long-acting. Interestingly, some research suggests that only 49% of adults are prescribed long-acting agents [114]. The proportion of adults on long-acting agents is considerably lower than that for children.

Non-stimulant options are also similar to pediatric ADHD options, although bupropion and modafinil are used more often in adults than in children with ADHD. However, the only non-stimulant with FDA approval for adults with ADHD is atomoxetine.

The potential for diversion and misuse may be greater in adults than in children, as parents might control the medications for the latter. Stimulant misuse seems to be more common in those with comorbid alcohol-, drug- and cigarette-related problems, and those with higher levels of ADHD symptoms [115]. In addition, long-acting stimulants are less likely to be misused or diverted than short-acting stimulants.

In pediatric ADHD, a combined treatment approach generally consists of pharmacotherapy and some form of psychosocial intervention, generally consisting of training parents in behavioral management, consultation with teachers/school personnel and individual work with the child [116]. For example, behavioral parent training (BPT) programs seem to be effective for children with disruptive behaviors, irrespective of co-occurring attentional/hyperactive difficulties. BPT techniques generally consist of training parents in general operant conditioning techniques such as contingent application of reinforcement or punishment in response to appropriate/inappropriate behaviors. Reinforcement procedures have typically relied on praise, privileges or tokens, whereas punishment methods have usually been loss of positive attention, privileges or tokens, or formal 'time out' from reinforcement.

In adults, a combined treatment approach similarly typically consists of pharmacotherapy and psychosocial intervention. However, unlike pediatric ADHD, there is some evidence that CBT interventions are efficacious (please see Table 3 for list of common non-pharmacological interventions for managing ADHD).

**Table 3:** Non-pharmacological treatments for attention deficity hyperactivity disorder (ADHD)

Name	Description
Parent training in behavior management	A training intervention is to gather a detailed accounting of behavioral problems including when and in what situations misbehaviors occur. It is also useful to record how parents and other adults react to the behaviors, and what subsequent interactions take place as a result of those reactions. In sum, what are the social contingencies that might be cueing, exacerbating or sustaining inappropriate behavior, if any? What disciplinary methods are used in the home now and in the past, and what formalized help have parents sought and obtained for managing the problems? Both parents need to be involved if both have contact with the child. At the very least, the non-attending parent must be supportive of the one attending training if the transfer of skills from the group to the home setting is to be enhanced. If others regularly care for the child, they may also be involved in the training, so that the child experiences consistency across the routine caregivers in his life.
School interventions	Often include alterations to the curriculum and workload to better mesh with the limited attention, persistence and disorganization of the child with ADHD; special educational services ('push-in' or mainstreaming assistance to regular teachers; 'pull-out' services to focus on more individualized child training, self-contained classes); increases in sources of positive reinforcement for work productivity; occasional use of immediate and systematic negative consequences for disruptive or inappropriate behavior; implementation of a daily school behavior report card (the ratings on which are linked to a home token economy), peer-tutoring or other innovative approaches to using peer influence to achieve classroom goals; and more communication with parents. In short, greater accountability of the child to teachers and others, including more immediate, frequent and salient feedback for performance, and increased structuring of the classroom environment and teaching materials have all been shown to benefit the child with ADHD in school.
Cognitive behavioral therapy	The emphasis on the process of restructuring or modifying an individual's thoughts to create behavioral effects is what differentiates CBT therapists from behavioral therapists. Aspects of CBT that differentiate the treatment from other therapeutic orientations such as psychodynamic therapy and interpersonal therapy include the following. • Use of homework and outside-of-session activities. • The CBT therapist is more active and directs session activity. • CBT focus is clearly on the future. • The CBT therapist explicitly teaches skills for coping with symptoms. • The CBT therapist focuses on cognitive experiences (especially dysfunctional thoughts and beliefs). • The therapist provides explicit information to the patient about the treatment, the disorder and its symptoms.

CBT joins together cognitive and behavioral therapies, and gained popularity in the late 1960s as a treatment approach. Cognitive therapists believe that how a person interprets an event is more important than the actual event itself. Therefore, treatment focuses more on cognitions than on overt behaviors; reducing dysfunctional thoughts helps to improve adjustment [117]. Behavioral models emphasize the role of basic learning principles (operant conditioning, classic conditioning, observational learning) in developing and maintaining behavior, both adaptive and maladaptive. Rather than focusing on cognitions, behavioral therapy spotlights the stimuli and contingencies that maintain maladaptive behaviors. Treatments that are cognitive-behavioral in nature include both cognitive and behavioral procedures and have at their core three fundamental beliefs [118]: 1) cognitive activity affects behavior; 2) cognitive activity can be monitored and modified and 3) behavioral change can be produced by cognitive change.

Safren *et al. *developed a CBT program for adults with ADHD as a supplement to their medication treatment [119]. A recently published study suggests that, when compared with adults with ADHD who received relaxation training and educational support, adults with ADHD who received 12 sessions of CBT had lower self-reported ADHD symptoms and greater functional improvements as rated by a blinded assessor [119]. There were more treatment responders in the CBT group (53%) relative to the relaxation training and educational support group (23%), and gains were maintained over periods of 6 and 12 months [119].

Bramham *et al. *also developed a group CBT workshop program [120]. Using three 1-day workshops held monthly, six sessions were included in the program. The results suggested that, relative to the controls, adults with ADHD who participated in the workshop increased their knowledge of ADHD. However, less optimistically, one-third of participants dropped out during the course of the CBT workshop. Furthermore, both groups reported less depression and anxiety. Both groups also had improved self-esteem at the end of the study, but the intervention group reported a greater improvement in self-esteem [120]. Others have also recently developed group interventions relying on CBT strategies [121].

In sum, CBT seems to be a promising treatment approach to complement pharmacotherapy. At this point, there is limited evidence that CBT is efficacious on its own. However, when added to pharmacotherapy, emerging evidence suggests CBT improves treatment outcomes compared with medications alone.

#### *Challenges of treating ADHD*

There are several challenges associated with the treatment of people with ADHD. The first challenge is related to the clinical complexity of the cases themselves; the vast majority of people with ADHD, both child and adult, have a comorbid psychiatric disorder [122-125]. Thus, even relatively successful treatment of the ADHD symptoms may be associated with only modest functional improvements in the real world.

In the presence of significant comorbidity, complex combined treatments may be required, and the results may be frustrating. Diligent attempts to clarify the co-occurring conditions and related features (for example, poor social skills, low academic abilities become essential in cases resistant to treatment. Although most people with ADHD will respond favorably to pharmacological interventions [126], optimal functioning may not be attained. Even in non-comorbid ADHD cases, optimal functioning occurs in only roughly one in four children with ADHD [127,128]. Most patients show residual disabilities in several areas, including executive functioning, deficient emotional self-regulation, and 'real-world' functioning in school or employment, or in maintaining relationships. Some have suggested that ADHD is a disorder of performance, not knowledge [129]. Thus, despite reduced ADHD symptoms and knowing how best to manage their affairs, residual impulsivity often continues to negatively affect functioning. For this reason, establishing reasonable expectations with patients and parents may be crucial for the success and continuity of the treatment. Likewise, medications may improve some but not all aspects of cognitive functioning, and even when both symptoms and cognitive function are improved, the two are not necessarily correlated [130].

A second challenge in treating ADHD is related to the methods of treatment, and the optimization of the risk:benefit ratio. Optimization of the treatment response often requires careful adjustments in doses and particular distributions of the doses during the day to maximize the effect of medications at the point of performance. Combined pharmacotherapy (for example,, antidepressant plus stimulant for ADHD and comorbid depression) is often needed for patients with comorbid disorders, and is sometimes indicated when ADHD is the only presenting problem. Adjunct psychosocial treatments are often useful, but these should be targeted to patients based on a needs assessment.

Possibly as a function of the disorder itself, non-adherence to treatment regimens is high in ADHD [131]. In addition to the disorganization inherent in the disorder, other contributors to poor treatment adherence may be denial, externalization of the problem and medication side-effects [131]. Little is known about predictors of long-term adherence, so more work is needed to improve this crucial component of treatment efficacy.

In sum, despite considerable advances in our understanding and treating ADHD, the disorder remains difficult to manage. Poor treatment adherence and psychiatric comorbidity clearly complicate treatment and negatively affect outcomes.

## Conclusions

Cognitive neuroscience has permitted a greater understanding of ADHD. Recent research and novel drug developments have provided new treatment options for adolescents and adults with ADHD. New stimulant formulations have made it possible to tailor treatment to the duration of efficacy required by patients and to help mitigate the potential for abuse, misuse and diversion. Although they tend to be less efficacious than stimulants, new non-stimulant options also allow for extended duration of treatment without the adverse consequences associated with stimulant therapy. Progress in non-medical therapies now provides several options for patients who cannot or will not use medications, and for the many medication-treated patients who continue to show residual disability.

Looking toward the future, research will need to address several unmet needs. Many treated people with ADHD continue to have problems with executive functioning and deficient emotional self-regulation. These problems persist in many patients even when the core ADHD symptoms (as outlined in the *Diagnostic and Statistical Manual of Mental Disorders, Fourth edition (*DSM-IV)) are effectively treated. Future treatment development should aim at developing both psychosocial and medical treatments for these areas of difficulty. Future treatment research should also work to define and achieve optimal treatment outcomes for people with ADHD. Although current treatments are effective for achieving substantial symptom reduction in most patients, more work is needed to achieve full symptom reduction, and to reduce the burden of ADHD-associated disabilities.

There are also diagnostic challenges for clinicians that could be addressed by future research. ADHD symptoms, especially hyperactive-impulsive symptoms, tend to decline through adolescence into adulthood, so that the adult presentation of ADHD differs somewhat from the childhood presentation. Helping clinicians understand these differences, and how such differences should affect the application of diagnostic criteria requires more work.

Ideally, medical and psychological treatments should be tailored to the underlying pathophysiology of the patient. Theoretically, this should be possible by using the scientific literature on the neurobiology of ADHD with treatment outcome studies, as it is possible that patients with specific brain-imaging abnormalities or genetic variants would have different responses to treatments. To date, most of this work has been done in the area of pharmacogenetics which, although promising, cannot yet guide treatment choices [132-134]

In summary, although the science of ADHD and its application to diagnosis and treatment have made great strides, more work is needed to improve the lives of patients and families affected by the disorder.

## List of Abbreviations

ADHD: attention deficit/hyperactivity disorder; CBT: cognitive behavioral therapy; LDX: lisdexamfetamine dimesylate; OROS: osmotic-release oral system; SNRI: Serotonin-norepinephrine reuptake inhibitor

## Competing interests

Kevin M. Antshel*, Teresa M. Hargrave, Mihai Simonescu, Prashant Kaul, Kaitlin Hendricks and Stephen V. Faraone

KMA, TMH, MH, PK, and KH report no biomedical financial interests or potential conflicts of interest. SVF has in the past year received consulting fees and has been on Advisory Boards for Eli Lilly, Ortho-McNeil and Shire Development and has received research support from Shire and the National Institutes of Health; in previous years, has received consulting fees or has been on advisory boards or has been a speaker for Shire, McNeil, Janssen, Novartis, Pfizer, Ortho-McNeil and Eli Lilly; in previous years has received research support from Eli Lilly, Shire, Pfizer and the National Institutes of Health, and has a published book with Guilford Press: *Straight Talk About Your Child's Mental Health*.

## Authors' contributions

All authors have been involved in drafting the manuscript or critically revising it or important intellectual content, and have given final approval of the version to be published.

## Pre-publication history

The pre-publication history for this paper can be accessed here:

http://www.biomedcentral.com/1741-7015/9/72/prepub
